# Engineered Synthetic STxB for Enhanced Cytosolic Delivery

**DOI:** 10.3390/cells12091291

**Published:** 2023-04-30

**Authors:** Justine Hadjerci, Anne Billet, Pascal Kessler, Gilles Mourier, Marine Ghazarian, Anthony Gonzalez, Christian Wunder, Nesrine Mabrouk, Eric Tartour, Denis Servent, Ludger Johannes

**Affiliations:** 1 Cellular and Chemical Biology Unit, Institut Curie, Université PSL, U1143 INSERM, UMR3666 CNRS, 26 Rue d’Ulm, CEDEX 05, 75248 Paris, France; 2 Université de Paris, 85 Boulevard Saint-Germain, 75006 Paris, France; 3 DMTS/SIMoS, CEA, Université Paris Saclay, 91191 Gif sur Yvette, France; 4 PARCC, INSERM, Université Paris Cité, 75015 Paris, France; 5 Department of Immunology, Hôpital Européen Georges-Pompidou, AP-HP, CEDEX 15, 75908 Paris, France

**Keywords:** synthetic carrier, organic synthesis, endosomal escape, hydrophobic moieties, engineered protein

## Abstract

Many molecular targets for cancer therapy are located in the cytosol. Therapeutic macromolecules are generally not able to spontaneously translocate across membranes to reach these cytosolic targets. Therefore a strong need exists for tools that enhance cytosolic delivery. Shiga toxin B-subunit (STxB) is used to deliver therapeutic principles to disease-relevant cells that express its receptor, the glycolipid Gb3. Based on its naturally existing membrane translocation capacity, STxB delivers antigens to the cytosol of Gb3-positive dendritic cells, leading to the induction of CD8^+^ T cells. Here, we have explored the possibility of further increasing the membrane translocation of STxB to enable other therapeutic applications. For this, our capacity to synthesize STxB chemically was exploited to introduce unnatural amino acids at different positions of the protein. These were then functionalized with hydrophobic entities to locally destabilize endosomal membranes. Intracellular trafficking of these functionalized STxB was measured by confocal microscopy and their cytosolic arrival with a recently developed highly robust, sensitive, and quantitative translocation assay. From different types of hydrophobic moieties that were linked to STxB, the most efficient configuration was determined. STxB translocation was increased by a factor of 2.5, paving the path for new biomedical opportunities.

## 1. Introduction

Delivery approaches of pharmaceutical compounds have already contributed to the development and commercialization of therapeutic principles [1]. However, the targeting of bioactive macromolecules, including peptides and nucleic acids, to their cytosolic targets remains an important bottleneck for therapy, as their spontaneous translocation across endosomal membranes (termed endosomal escape) is highly inefficient [2,3]. Hence, there is a need to develop performant vectors to enhance the delivery of bioactive macromolecules into the cytosol of therapeutically relevant target cells.

Based on its unique intracellular trafficking characteristics and ability to target dendritic cells (DCs), the B-subunit of Shiga toxin (STxB) has been developed as a vaccination tool. When antigens are delivered by STxB to the cytosol of DCs, a specific long-lasting CD8^+^ cytotoxic T lymphocyte response, including mucosal resident memory T cells is observed, which protects mice against tumor growth and viral infection [4,5,6,7,8,9,10,11,12]. The naturally existing endosomal escape capacity of STxB has previously been quantified at 0.47% of total cell-associated protein [13]. While this level is sufficient to induce efficient antigen cross-presentation [4,5,6,7,8,9,10,11,12], it may be too low for other applications.

Endosomal escape of enveloped viruses is often based on the destabilization of the lipid bilayer by insertion into the membrane of motifs containing hydrophobic amino acids [14]. Cell-penetrating peptides (CPPs) are another class of molecules known for their ability to cross the membrane barrier; these often contain hydrophobic domains [15,16,17]. The mechanism by which at least some CPPs translocate to the cytosol also involves perturbation and reorganization of the lipid bilayer. Inspired by these strategies to solve the translocation issue, Lönn et al. have shown that the conjugation of synthetic hydrophobic endosomal escape domains to the HIV TAT protein transduction module significantly enhances its cytosolic delivery [15].

We recently achieved the linear chemical synthesis and in vitro refolding of STxB [18]. Purely synthetic STxB behaves very similarly to the recombinant counterpart in terms of biophysical characteristics and intracellular trafficking. This chemical synthesis scheme has generated new opportunities for the engineering of STxB, e.g., the insertion of unnatural amino acids for bio-orthogonal conjugation.

Here, we developed an engineered synthetic STxB platform with an enhanced cytosolic delivery capacity based on hydrophobic substitutions. For that, we designed STxB variants that contained two chemical handles for site-specific functionalization. One handle was used to conjugate hydrophobic endosomal escape enhancers to the protein. The other one served in the current study for the coupling to a chemical cytosolic arrival reporter that we have recently developed [13]. By screening a small library of hydrophobic moieties with this setup, we succeeded in increasing STxB cytosolic arrival by a factor of 2.5. We discuss how the second handle would be used in biomedical applications for the conjugation of therapeutic entities.

## 2. Materials and Methods

### 2.1. UPLC-MS Analysis

Samples were analyzed with a Waters UPLC-MS system (Milford, MA, USA) comprised of an ACQUITY UPLC H-Class sample manager, an ACQUITY UPLC PDA eLambda Detector, and a Single Quadrupole Detector 2 for positive and negative electron spray ionization (ESI) mass spectra. An ACQUITY UPLC BEH C18, 1.7 μm 2.1 × 50 mm column, was used. Solvents were: A—0.1% formic acid in Milli-Q water and B—0.1% formic acid in acetonitrile. The flow rate was 0.6 mL/min. Gradients were: 0.2 min 5% B for accumulation at the head of the column, followed by 2.3 min linear gradient from 5% to 95% B. Compound purities were calculated by surface peak integration from UPLC analyses.

### 2.2. Synthetic STxB Variant Syntheses

STxB was synthesized and refolded as described elsewhere [18]. *N*-Fmoc-*N′*-(azido-PEG4)-L-lysine was purchased from Tebu-Bio (Le Perray-en-Yvelines, France). Except when mentioned otherwise, concentrations in this paper correspond to STxB pentamers. UPLC-MS analyses of STxB variants are presented in Supporting Information (Figure S1).

### 2.3. Cell Culture

NG-SNAP cells (monoclonal HeLa cells stably expressing the cytosolic mNeon-Green-SNAP-tag fusion protein, see [13] for more details) were cultured at 37 °C under 5% CO_2_ in Dulbecco’s modified Eagle’s medium (DMEM), supplemented with 10% fetal bovine serum, 0.01% penicillin–streptomycin, 4 mM glutamine, 1 mM pyruvate (complete medium), and geneticin (200 μg/mL).

### 2.4. Glycosphingolipid Depletion

Cells were pre-treated for seven days with 5 µM of PPMP (DL-threo-1-phenyl-2-palmitoylamino-3-morpholino-1-propanol) in DMEM with 0.01% penicillin–streptomycin, 4 mM glutamine, 1 mM pyruvate, 5% fetal bovine serum, and 200 µg/mL geneticin.

### 2.5. Intracellular Trafficking Assay

The day before, cells were seeded on glass lamellae in 4-well dishes, 80,000 cells/well. On the day of the experiment, cells were incubated for 30 min at 4 °C in 500 μL of 40 nM STxB in an ice-cold complete medium for binding, followed by three washes with PBS^++^ (PBS, 0.5 mM MgCl_2_, 1 mM CaCl_2_). Complete medium at 37 °C was added to cells, which were then incubated for 50 min at 37 °C for synchronized internalization. Cells were washed three times with PBS^++^, fixed with 4% PFA in PBS for 15 min, washed once with 50 mM NH_4_Cl, and incubated with 50 mM NH_4_Cl for 30 min to quench the PFA. Cells were washed three times with PBS/BSA/saponin (PBS/0.2% *w*/*v* bovine serum albumin/0.02% *w*/*v* saponin) and permeabilized at room temperature for 30 min in PBS/BSA/saponin. Lamellae were incubated on 30 μL of antibody dilution into PBS/BSA/saponin for 30 min at room temperature and then washed three times with PBS/BSA/saponin. Primary antibodies were a homemade mouse monoclonal anti-STxB antibody (13C4, used at 1/250 dilution) and a homemade rabbit polyclonal antibody against the Golgi marker giantin (used at 1/100 dilution). The secondary antibodies were Cy3-coupled anti-mouse and Alexa488-coupled anti-rabbit IgGs, used at 1/100 dilution each. Lamellae were washed in water and then deposited onto slides with 6 μL of Fluoromont G. Polymerization was performed for 30 min at 37 °C.

### 2.6. Microscopy

Images were recorded using the inverted Eclipse Ti-E (Nikon, Tokyo, Japan) with spinning disk CSU-X1 (Yokogawa, Tokyo, Japan) and 60× CFI Plan Apo. Stacks of 16 images at 0.2 μm depth were integrated with Metamorph software by Gataca Systems (Massy, France).

### 2.7. Syntheses of Hydrophobic Moieties

The following compounds, indicated by capital letters, are correspondingly tagged in later figures:

(A) DBCO–butyroyl: 13 mg DBCO-NH_2_ (Iris biotech, Marktredwitz, Germany, CAS: 1255942-06-3, MW 276.33 g/mol) was reacted with 1.2 eq butyryl chloride in the presence of 1 eq triethylamine (Et_3_N) in DCM under argon. The reaction was monitored by thin-layer chromatography (TLC) and was complete after 1 h. After evaporation, the product was purified by flash column chromatography with 2% MeOH in DCM, yielding 9.6 mg DBCO–butyroyl as a yellowish brown oil (yield 59%, purity 96%).

(B) DBCO–benzoyl: 12.1 mg DBCO-NH_2_ (Iris biotech, CAS: 1255942-06-3, MW 276.33 g/mol) was reacted with 1.2 eq benzoyl chloride in the presence of 1 eq Et_3_N in DCM under argon. The reaction was monitored by TLC and was stopped at 2 h. After evaporation, the product was purified by flash column chromatography with 2% MeOH in DCM, yielding 14.4 mg DBCO–benzoyl as a yellowish brown oil (yield 87%, purity 99%).

(C) DBCO–hexanoyl: 11 mg DBCO-NH_2_ (Iris biotech, CAS: 1255942-06-3, MW 276.33 g/mol) was reacted with 1.2 eq hexanoyl chloride in the presence of 1 eq Et_3_N in DCM under argon. The reaction was monitored by TLC and was complete after 2 h. After evaporation, the product was purified by flash column chromatography with 2% MeOH in DCM, yielding 9.1 mg DBCO–hexanoyl as a yellowish brown oil (yield 61%, purity 90%).

(D) DBCO-PEG4–butyroyl: 10.3 mg DBCO-PEG4-NH_2_ (Iris biotech, CAS: 1255942-08-5, MW 523.62 g/mol) was reacted with 1.2 eq butyryl chloride in the presence of 1 eq Et_3_N in DCM under argon. The reaction was monitored by TLC and was stopped after 7 h. Following evaporation, the product was purified by flash column chromatography with a gradient of 1 to 8% MeOH in DCM, yielding 4.4 mg DBCO-PEG4–butyroyl as a colorless oil (yield 38%, purity 99%).

(E) DBCO-PEG4–benzoyl: 10.3 mg DBCO-PEG4-NH_2_ (Iris biotech, CAS: 1255942-08-5, MW 523.62 g/mol) was reacted with 1.2 eq benzoyl chloride in the presence of 1 eq Et_3_N in DCM under argon. The reaction was monitored by TLC and was stopped after 7 h. Following evaporation, the product was purified by flash column chromatography with a gradient of 1 to 8% MeOH in DCM, yielding 1.9 mg DBCO-PEG4–benzoyl as a colorless oil (yield 15%, purity 93%).

(F) DBCO-PEG4–octanoyl: 10.0 mg DBCO-PEG4-NH_2_ (Iris biotech, CAS: 1255942-08-5, MW 523.62 g/mol) was reacted with 1.2 eq octanoyl chloride in the presence of 2 eq Et_3_N in DCM under argon. The reaction was monitored by TLC and was stopped after 5 h. Following evaporation, the product was purified by flash column chromatography with a gradient of 1 to 6% MeOH in DCM, yielding 1.2 mg DBCO-PEG4–octanoyl as a brown oil (yield 10%, purity 81%).

(G) DBCO-PEG4-2–naphtoyl: 10.2 mg DBCO-PEG4-NH_2_ (Iris biotech, CAS: 1255942-08-5, MW 523.62 g/mol) was reacted with 1.2 eq 2-naphthoyl chloride in the presence of 1 eq Et_3_N in DCM under argon. The reaction was monitored by TLC and was stopped after 5 h. Following evaporation, the product was purified by flash column chromatography with a gradient of 1 to 6% MeOH in DCM, yielding 6.0 mg DBCO-PEG4-2–naphtoyl as a brown oil (yield 45%, purity 95%).

UPLC-MS analyses of hydrophobic moieties are presented in Supporting Information (Figure S2).

### 2.8. Double Conjugations

A total of 40 µM of synthetic STxB double variants, or of recombinant rSTxB(70C) produced as previously described [19], were conjugated to 1.2 eq maleimide–benzylguanine–biotin (called BG–biotin, see [13] for synthesis) for 4 h at 21 °C, 750 rpm in PBS (0.14 M NaCl, 2.7 mM KCl, 0.01 M phosphate buffer pH 7.4) with 5% DMSO, followed by the addition of 2 eq of hydrophobic moieties in DMSO (final DMSO volume in the reaction: 10%) and overnight reaction at 21 °C, 750 rpm. Excess BG–biotin and hydrophobic moieties were removed on two Zeba spin desalting columns (0.5 mL; 7K MWCO; Thermo Fisher Scientific, Asnières-sur-Seine, France), equilibrated in PBS. Double conjugate formation and the absence of remaining free BG–biotin or cyclooctyne-hydrophobic moiety after purification were validated by UPLC-MS.

### 2.9. Concentration Assessment of the Double Conjugates

To avoid the determination of the molar extinction coefficient at 280 nm, which changes between conjugates, we took advantage of the biotin present on the double conjugates for the measurement of the concentration by western blot using fluorescently labeled streptavidin. For that, samples were denatured in sample buffer 1× (62.5 mM Tris HCl pH 6.2% SDS, 10% glycerol, 0.1 mg/mL phenol red, 42 mM DTT) by heating for 5 min at 95 °C. Denatured conjugates, denatured standard STxB(70C)-(BG–biotin) (gel loading: 100 ng, 200 ng, 400 ng, and 600 ng), and molecular weight marker (Page Ruler Plus Prestained Protein Ladder from Thermo Fisher Scientific, Asnières-sur-Seine, France) were run at 90 V on 4–20% Mini Protean TGX precast gels from Biorad in 1× Tris/glycine/SDS buffer from Biorad. Proteins were then transferred for 5 min onto 0.2 µm AmershamTM Protran^®^ nitrocellulose blotting membrane, using a Pierce G2 Fast Blotter instrument and 1-Step Transfer Buffer from Thermo Fisher Scientific (Asnières-sur-Seine France). Membranes were blocked at room temperature for 30 min in blocking buffer: 5% defatted milk in TBS-Tween (50 mM Tris, 150 mM NaCl, 0.1% Tween 20, pH 7.4). Protected from light, membranes were incubated for 1 h at room temperature with streptavidin Alexa 647 (Thermo Fisher Scientific, Asnières-sur-Seine, France, stock at 2 mg/mL in PBS) at 1/1000 dilution in blocking buffer and then washed three times for 5 min with TBS-Tween. Membranes were imaged using a ChemiDoc imaging system (Bio-Rad, Hercules, CA, USA) (Figure S3). Band intensity measurements and quantification of double conjugate concentrations were performed using Image Lab 6.1 software (Bio-Rad, Hercules, CA, USA).

### 2.10. Size Exclusion Chromatography

After 10 min of centrifugation at 17,000× *g*, STxB samples in PBS were analyzed on a Superdex™ 75 Increase 3.2/300 column (GE Healthcare, Uppsala, Sweden) equilibrated with PBS. The flow rate was 0.1 mL/min. A total of 10 to 20 uL were injected with sample concentrations between 0.06 and 0.6 mg/mL.

### 2.11. Relative Quantification of Membrane Translocation to the Cytosol

See [13] for a detailed protocol. Cells were incubated with 40 nM of STxB double conjugates for 3 h.

### 2.12. Data Analysis and Figures

Prism software (GraphPad, Boston, MA; USA) was used for statistical analysis and graph plotting, Fiji ImageJ software (National Institutes of Health, Bethesda, MD, USA) [20] for microscopy, Image Lab 6.1 for western blot image processing, Affinity Publisher software (v.2) to draw figures, and ChemDraw software (v.14) (PerkinElmer Informatics, Waltham, MA, USA) to draw chemical structures.

## 3. Results

### 3.1. Synthetic Double Variants of STxB

**Figure 1:**
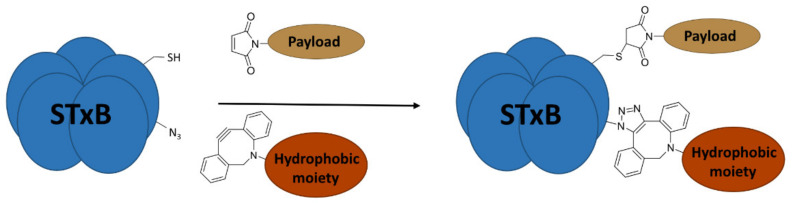
Experimental strategy. The sulfhydryl (SH) from C-terminal cysteine was used for coupling to payloads, here the membrane translocation reporter. The azide (N_3_) from unnatural amino acids was used for conjugation with hydrophobic entities. Up to five payloads and hydrophobic moieties were conjugated to each STxB homopentamer.

We have recently developed an approach to chemically synthesize STxB monomers and fold these into fully functional homopentamers [18]. Based on this, we set out to generate STxB variants with two functional groups for site-directed substitutions (Figure 1). For that, azido-containing amino acids were used as the first functionalization site for copper-free click chemistry-based coupling to hydrophobic endosomal escape enhancers. The second functionalization site was a C-terminal cysteine for sulfhydryl chemistry-based coupling to a reporter moiety for the determination of the arrival into the cytosol of corresponding STxB variants [13]. We had indeed found that synthetic STxB could be modified by azido-containing amino acids at several conjugation-accessible sites while remaining fully active [18]. Furthermore, the addition of a C-terminal cysteine was previously equally shown to yield a functional protein that can be coupled to different types of payloads [8,11,21,22,23,24,25,26].

Four positions on STxB were selected to place azides at variable distances from the membrane-binding face (Table 1). The corresponding four double variants containing the (70C) residue were then synthesized with azido lysines to replace amino acids D3, E10, H58, or N59. Another double variant was synthesized with an azido-PEG4-lysine at position N59. Position 59 is at 11 Å from the membrane, and PEG4 has an extended length of 14 Å.

All double variants had synthesis and folding yields that were very similar to mono-modified STxB(70C) (Table 1).

### 3.2. Hydrophobic Moieties

**Figure 2:**
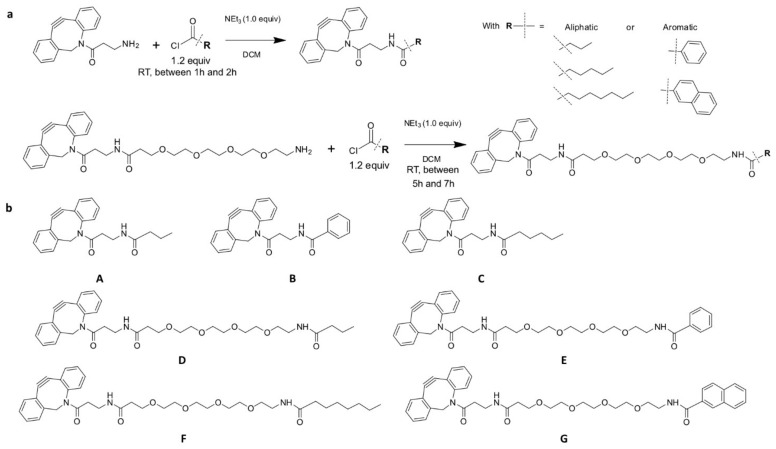
Syntheses of hydrophobic moieties. (**a**) Reaction schemes of DBCO-NH_2_ and DBCO-PEG4-NH_2_ coupling to acyl chloride-modified hydrophobic moieties. (**b**) Presentation of the seven hydrophobic moieties that were used for our study. For some moieties, the hydrophobic part was located close to DBCO (A, B, C). For others (D, E, F, G), PEG4 linkers were present between DBCO and hydrophobic parts.

The translocation enhancers were small aliphatic or aromatic moieties that were connected directly or via a PEG4 linker to a cyclooctyne for the conjugation to azide-containing STxB double variants (Figure 2a). A panel of seven hydrophobic moieties was synthesized with different levels of hydrophobicity as defined by the length of the aliphatic chain (from 4 to 8 carbons) or the number (1 or 2) of aromatic cycles (Figure 2b, corresponding UPLC-MS analyses in Figure S2). 

STxB double variants were also modified at the C-terminal cysteine by the translocation reporter molecule maleimide–benzylguanine–biotin (BG–biotin) for the Cyto-SNAP membrane translocation assay [13]. Both conjugation steps were performed with translocation reporter and hydrophobic moieties in excess to push double conjugation as much as possible to completion. The Cyto-SNAP assay is based on the modification of STxB with a translocation reporter containing a benzylguanine and a biotin moiety. The cytosolic delivery of modified STxB is evaluated using a specific cell line that expresses the SNAP-tag-mNeonGreen fusion protein in the cytosol. STxB is incubated for a few hours with the SNAP-tag-mNeonGreen cell line. Once STxB reaches the cytosol of the cells, it encounters the fusion protein. The benzylguanine on the translocation reporter of STxB covalently reacts with the SNAP-tag. The cytosolic fraction of the carrier is isolated using beads coated with a mNeonGreen antibody after cell lysis. In order to block unreacted SNAP-tag, the cells are incubated with SNAP-cell block solution just before the cell lysis. Finally, streptavidin-horseradish peroxidase (HRP) is used for the quantitative detection of biotin on the STxB-reporter. The quantity of cytosolic STxB is linked to the sample absorbance after the reaction of HRP with o-phenylenediamine (OPD), forming 2,3-diaminophenazine, which has a yellow color (ELISA).

Solubility issues were encountered with increasing hydrophobicity, as reflected by the yields of conjugated protein after purification (Table S1). The concentrations of engineered STxB were determined by western blotting with fluorophore-modified streptavidin that recognized the biotin from the translocation reporter (Figure S3).

### 3.3. Membrane Translocation of Engineered STxB

**Figure 3:**
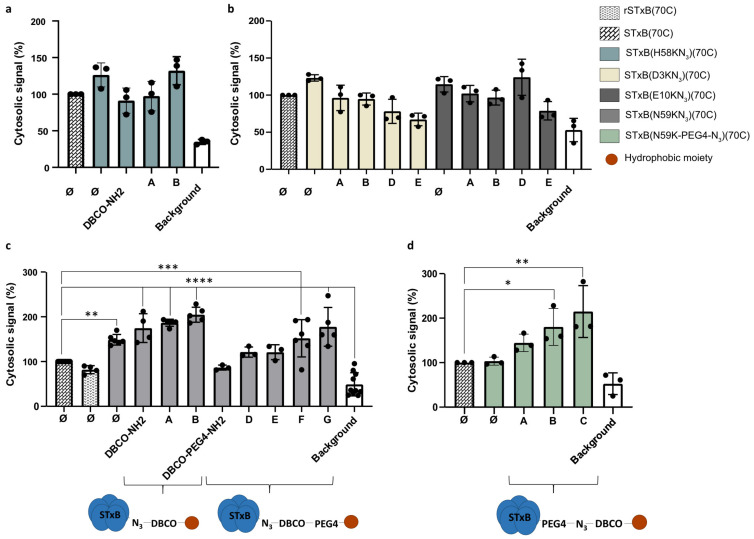
Relative comparisons of membrane translocation of engineered STxB variants to the cytosol, using the Cyto-SNAP assay. Capital letters correspond to the hydrophobic moiety conjugated to STxB (from A to G as presented in Figure 2, Ø: no hydrophobic moiety). In all experiments, the cytosolic signal is expressed as a percentage of the signal obtained for the synthetic STxB(70C) control. (**a**) Comparison of aliphatic and aromatic hydrophobic moieties conjugated to the variant STxB(H58KN_3_)(70C). No significant increase in cytosolic signal was observed. (**b**) Comparison of aliphatic and aromatic hydrophobic moieties with or without PEG4 linker conjugated to the variants STxB(D3KN_3_)(70C) and STxB(E10KN_3_)(70C). No significant increase in cytosolic signal was observed, even with a PEG4 linker. (**c**) Comparison of a set of hydrophobic moieties conjugated to the variant STxB(N59KN_3_)(70C). The translocation capacity of STxB increased with the hydrophobicity of the moieties. (**d**) Comparison of hydrophobic moieties conjugated to the variant STxB(N59K-PEG4-N_3_)(70C). Positioning the PEG linker between STxB and the azide increased STxB translocation compared to its localization between the DBCO and the hydrophobic moiety. * *p* < 0.05, ** *p* < 0.005, *** *p* ≤ 0.0005, **** *p* < 0.0001.

The cytosolic arrival of engineered STxB variants was investigated using the Cyto-SNAP assay [13]. When comparing the four amino acid positions of STxB onto which the hydrophobic moieties were coupled, it became apparent that only one, i.e., N59, yielded a substantial increase in membrane translocation capacity (Figure 3).

Surprisingly, this positive effect was not observed for the neighboring position H58 (Figure 3a). We interpret this finding with the fact that H58 is directly oriented onto the membrane interaction surface of STxB such that the hydrophobic moieties may have interfered with the binding to Gb3 receptors.

For the D3 and E10 positions, we suggest that the absence of a stimulatory effect on membrane translocation upon substitution with hydrophobic moieties (Figure 3b) may have originated from the fact that even with a PEG4 linker, these were localized too far from the membrane, i.e., respectively 14 Å and 20 Å.

For the STxB(N59KN_3_)(70C) double variant, the baseline of translocation to the cytosol was already increased when compared to recombinant or synthetic STxB(70C) (Figure 3c). Different hydrophobic moieties, as shown in Figure 2, were then compared. The general trend was that translocation to the cytosol was progressively enhanced with increasing hydrophobicity (Figure 3c). The addition of the PEG4 linker between the azide on STxB(N59KN_3_)(70C) and the hydrophobic moieties decreased translocation efficiency while keeping the same trend with increasing hydrophobicity (Figure 3c). Notably, translocation efficiency increased when the PEG4 linker was located between STxB and the azide (Figure 3d). Indeed, even if the distance between the hydrophobic moiety and the membrane was equivalent, in the second case, the hydrophobicity of the DBCO and the hydrophobic moiety were gathered. With some configurations, especially when benzoyl was used as a translocation enhancer with the variant STxB(N59KN_3_)(70C), the cytosolic delivery was enhanced 2-fold when compared to synthetic STxB(70C) and 2.5-fold when compared to the recombinant rSTxB(70C) (corresponding analysis Figure S4).

### 3.4. Gb3 Binding and Intracellular Trafficking

**Figure 4:**
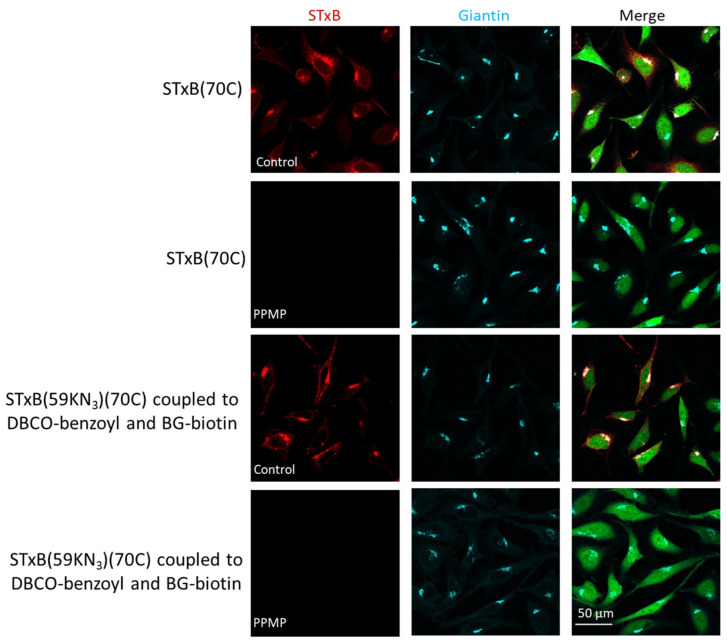
Targeting Gb3-expressing cells by engineered STxB. Intracellular trafficking for 50 min at 37 °C of 40 nM STxB variants on NG-SNAP cells under the indicated conditions. Merge: STxB channel in red, Golgi channel in cyan, and cytosolic mNeonGreen in green. Membrane translocation-engineered STxB colocalized with the Golgi only in Gb3 expressing NG-SNAP cells.

Wild-type STxB specifically targets Gb3-expressing cells [27] and undergoes retrograde transport from the plasma membrane to the Golgi apparatus [28]. We, therefore, set out to test whether these characteristics were preserved for membrane translocation-enhanced STxB variants. In NG-SNAP cells, both STxB(70C) and STxB(N59KN_3_)(70C) coupled to DBCO–benzoyl and BG–biotin extensively colocalizes with the Golgi apparatus upon incubation for 50 min at 37 °C with NG-SNAP HeLa cells (Figure 4). This demonstrated that the intracellular trafficking characteristics of the protein were not perturbed by these substitutions. Furthermore, we found that none of these two proteins bound to and were internalized into NG-SNAP cells from which glycosphingolipids were depleted using PPMP (Figure 4). It thereby clearly appeared that also the Gb3 binding specificity of STxB was not altered by its substitution with the endosomal escape enhancer.

These unperturbed trafficking characteristics strongly suggested that the STxB structure was preserved, even after conjugation to hydrophobic moieties. Previous studies have indeed shown that STxB exists either as a folded pentamer or as an unfolded monomer, which would be insoluble in an aqueous solution and unable to undergo retrograde trafficking [29,30,31]. Consistently, we observed by size exclusion chromatography that wild-type synthetic and recombinant STxB, the synthetic STxB(N59KN_3_)(70C) double variant and the STxB(N59KN_3_-DBCO–benzoyl)(70C) conjugate all eluted as one single peak (Figure S5). For the latter the retention time was slightly increased, which was likely due to non-specific hydrophobic interactions of the benzoyl moiety with the column (Table S2).

## 4. Discussion

In this study, the backbone of STxB was used as a biological starting material for protein engineering. STxB double variants were synthesized with high yields. These were fully functional and eluted from gel filtration columns as a single peak, which documented the structural robustness of STxB.

A 2.5-fold increase in cytosolic arrival was achieved for engineered synthetic STxB compared to recombinant STxB. These results were comparable to published studies using the reversible esterification of proteins to increase their cytosolic delivery [32,33]. However, in these studies, the described esterifications were not site-specific and occurred at different positions (carboxylic acids on Asp and Glu residues as well as on the C-terminus), leading to heterogeneous conjugates. Our conjugation strategy was based on site-specific reactions, which allowed the production of homogeneous conjugates. It is expected that these conjugates will have enhanced pharmacokinetics and pharmacodynamics profiles.

Moreover, the possibility of choosing functionalization sites has enabled us to study the influence of their localization on the enhancement of translocation to the cytosol. Among the STxB double variants that were tested, only hydrophobicity modification of position 59 increased the translocation capacity of STxB. Very clearly, the possibility to synthesize STxB and modify the protein has been critical for the success of the current study and will be instrumental for future developments.

Because of solubility issues leading to protein precipitation, limitations were encountered as to the degree of additional hydrophobicity that could be added onto STxB. It has been observed that unmodified STxB binds better to hydrophobic interaction chromatography columns than expected from its pKi, suggesting that some hydrophobicity might already be present on its surface. For instance, Tyr 11, Phe 30, and Trp 34 are solvent-exposed. These might explain the endogenous membrane translocation capacity of STxB.

With its ability to target Gb3 receptors that are overexpressed by DCs, membrane translocation-optimized STxB might be a carrier of interest for the delivery of therapeutic compounds to DCs. Moreover, as STxB showed robustness to undergo modifications in its sequence without affecting its structure and functionality, it might be envisaged to introduce a third handle onto STxB. Thus, the co-delivery of several bioactive molecules to the cytosol of DCs would be possible using the same STxB platform. This kind of completely synthetic delivery platform would be of great interest in the context of anti-cancer immunotherapy, for instance, to concomitantly deliver tumoral antigens and siRNAs to interfere with the expression of immune checkpoint inhibitors. Indeed, the development of anti-cancer vaccines has known clinical failures mostly due to trials done for metastatic diseases, in which immunosuppressor mechanisms are predominant. The combination of immunomodulators that counteract this inhibition is expected to improve the efficacy of anti-cancer vaccines [34].

## Figures and Tables

**Table 1:** STxB variants synthesis yields and functionality.

STxB Variant	Positions of the Azide (Red) and Extra Cysteine (Orange)	Azide—Membrane Distance (Å)	Fmoc Synthesis yield (%)	Oxidation and Folding Yield (%)	Overall Yield (%)	Retrograde Trafficking
STxB(70C)	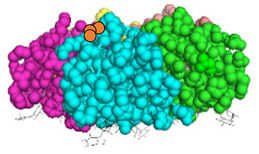	_	43	28	12.0	Functional
STxB(D3KN_3_)(70C)	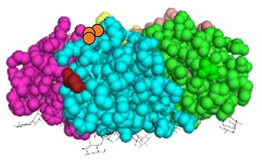	14	54	25	13.5	Functional
STxB(E10KN_3_)(70C)	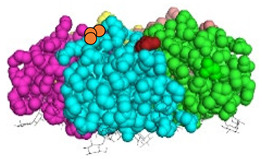	20	52	21	10.9	Functional
STxB(H58KN_3_)(70C)	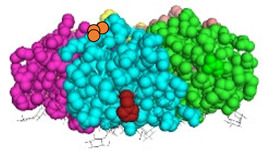	10	47	23	10.8	Functional
STxB(N59KN_3_)(70C)	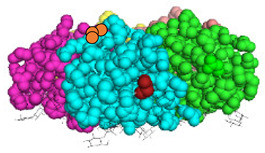	11	51	19	9.7	Functional
STxB(N59K-PEG4-N_3_)(70C)	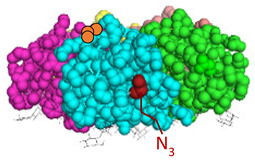	0	47	31	14.6	Functional

## Data Availability

All data needed to evaluate the conclusions in the paper are present in the paper and/or the Supplementary Materials. Additional data related to this paper may be requested from the authors.

## Supplementary Materials

The following supporting information can be downloaded at: https://www.mdpi.com/article/10.3390/cells12091291/s1. Figure S1: UPLC-MS analyses of refolded STxB variants; Figure S2: UPLC-MS analyses of hydrophobic moieties; Figure S3: Quantification of the concentrations of STxB double conjugates by western blotting; Figure S4: UPLC-MS analysis of STxB(N59KN3)(70C) conjugated to DBCO–benzoyl and BG–biotin; Figure S5: Size exclusion chromatography with STxB variants; Table S1: Double conjugation yields for coupling of hydrophobic moieties and BG–biotin to STxB double variants; Table S2: Size exclusion chromatography retention times for STxB variants.
