# Supplementary material for: Engineered Synthetic STxB for Enhanced Cytosolic Delivery
Source: Cells. 2023 Apr 30;12(9):1291. doi: 10.3390/cells12091291 (PMC10177378; doi:10.3390/cells12091291)
Supplement: Supplementary file 1 [file cells-12-01291-s001.zip › cells-2190515-supplementary.pdf]

Supporting Information

Engineered synthetic STxB for enhanced cytosolic delivery

Justine Hadjerci [1], Anne Billet [1, 2], Pascal Kessler [3], Gilles Mourier [3], Marine Ghazarian [3], Anthony Gonzalez [3], Christian Wunder [1], Nesrine Mabrouk [4], Eric Tartour [4, 5], Denis Servent [3], Ludger Johannes [1, *]

Table of contents

Figure S1. UPLC-MS analyses of refolded STxB variants. 2

Figure S2. UPLC-MS analyses of hydrophobic moieties. 3

Figure S3. Quantification of the concentrations of STxB double conjugates by western blotting. 4

Table S1. Double conjugation yields for coupling of hydrophobic moieties and BG-Biotin to STxB double variants. 4

Figure S4. UPLC-MS analysis of STxB(N59KN$_3$)(70C) conjugated to DBCO-benzoyl and BG-Biotin. 4

Figure S5. Size exclusion chromatography with STxB variants. 5

Table S2. Size exclusion chromatography retention times for STxB variants. 5

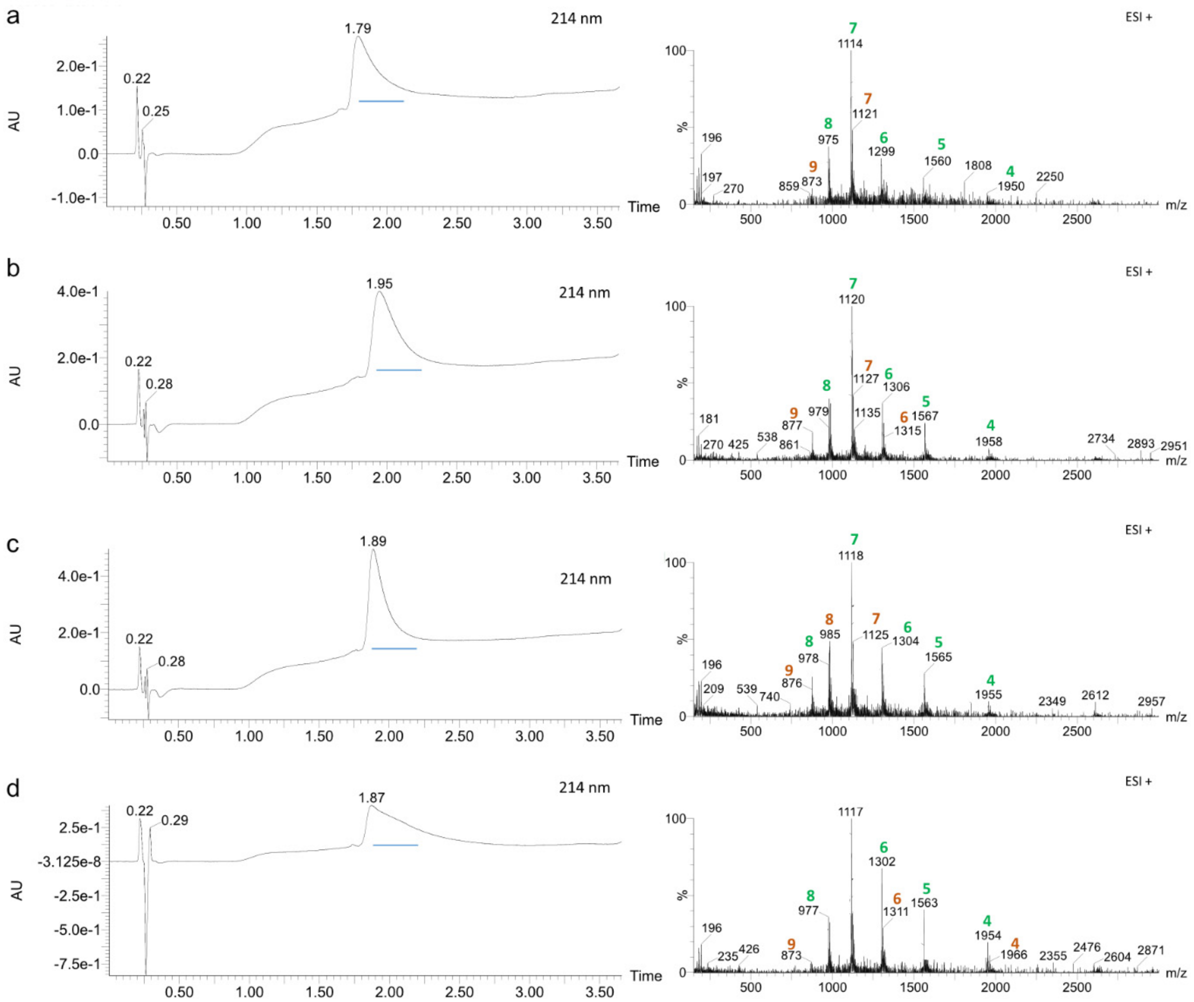

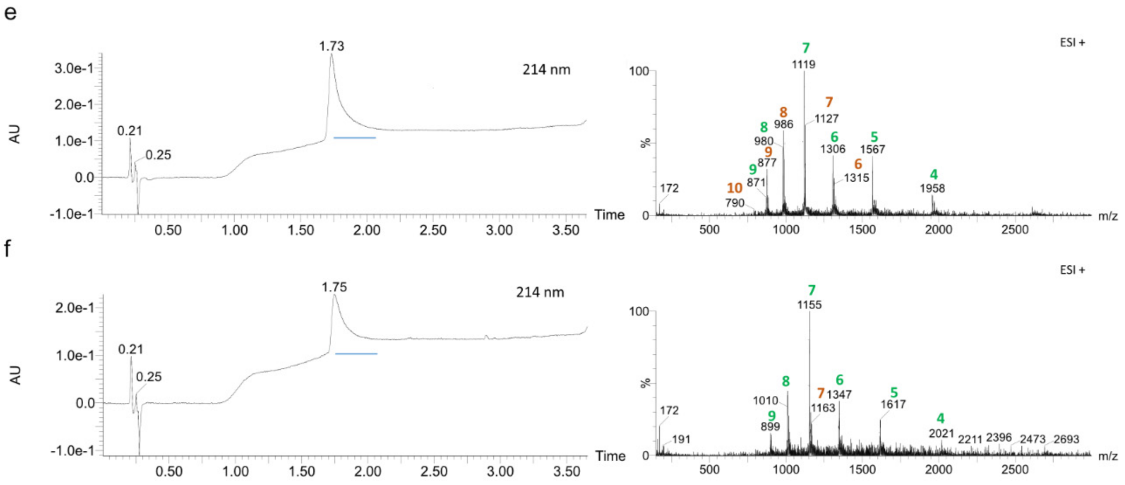

Figure S1. UPLC-MS analyses of refolded STxB variants.
Peaks corresponding to the expected products are annotated with their corresponding positive charge in green. Peaks annotated in orange correspond to monomers with a *t*-butyl adduct on the additional cysteine.
a) STxB(70C) MS m/z $C_{342}H_{533}N_{91}O_{109}S_4$ $[M+4H^+]^{4+}$ calculated: 1949.1; $[M+5H^+]^{5+}$ calculated: 1559.3; $[M+6H^+]^{6+}$ calculated: 1299.6; $[M+7H^+]^{7+}$ calculated: 1114.1; $[M+8H^+]^{8+}$ calculated: 975.1.
b) STxB(D3KN3)(70C) MS m/z $C_{344}H_{538}N_{94}O_{107}S_4$ $[M+4H^+]^{4+}$ calculated: 1958.7; $[M+5H^+]^{5+}$ calculated: 1567.1; $[M+6H^+]^{6+}$ calculated: 1306.1; $[M+7H^+]^{7+}$ calculated: 1119.7; $[M+8H^+]^{8+}$ calculated: 979.9.
c) STxB(E10KN3)(70C) MS m/z $C_{343}H_{536}N_{94}O_{107}S_4$ $[M+4H^+]^{4+}$ calculated: 1955.2; $[M+5H^+]^{5+}$ calculated: 1564.4; $[M+6H^+]^{6+}$ calculated: 1303.8; $[M+7H^+]^{7+}$ calculated: 1117.7; $[M+8H^+]^{8+}$ calculated: 978.1.
d) STxB(H58KN3)(70C) MS m/z $C_{342}H_{536}N_{92}O_{109}S_4$ $[M+4H^+]^{4+}$ calculated: 1953.2; $[M+5H^+]^{5+}$ calculated: 1562.7; $[M+6H^+]^{6+}$ calculated: 1302.5; $[M+7H^+]^{7+}$ calculated: 1116.5; $[M+8H^+]^{8+}$ calculated: 977.1.
e) STxB(N59KN3)(70C) MS m/z $C_{344}H_{537}N_{93}O_{108}S_4$ $[M+4H^+]^{4+}$ calculated: 1959.0; $[M+5H^+]^{5+}$ calculated: 1567.4; $[M+6H^+]^{6+}$ calculated: 1306.3; $[M+7H^+]^{7+}$ calculated: 1119.8; $[M+8H^+]^{8+}$ calculated: 980.0; $[M+9H^+]^{9+}$ calculated: 871.2.
f) STxB(N59K-PEG4-N3)(70C) MS m/z $C_{355}H_{558}N_{94}O_{113}S_4$ $[M+4H^+]^{4+}$ calculated: 2020.8 $[M+5H^+]^{5+}$ calculated: 1616.8; $[M+6H^+]^{6+}$ calculated: 1347.5; $[M+7H^+]^{7+}$ calculated: 1155.2; $[M+8H^+]^{8+}$ calculated: 1010.9; $[M+9H^+]^{9+}$ calculated: 898.7.

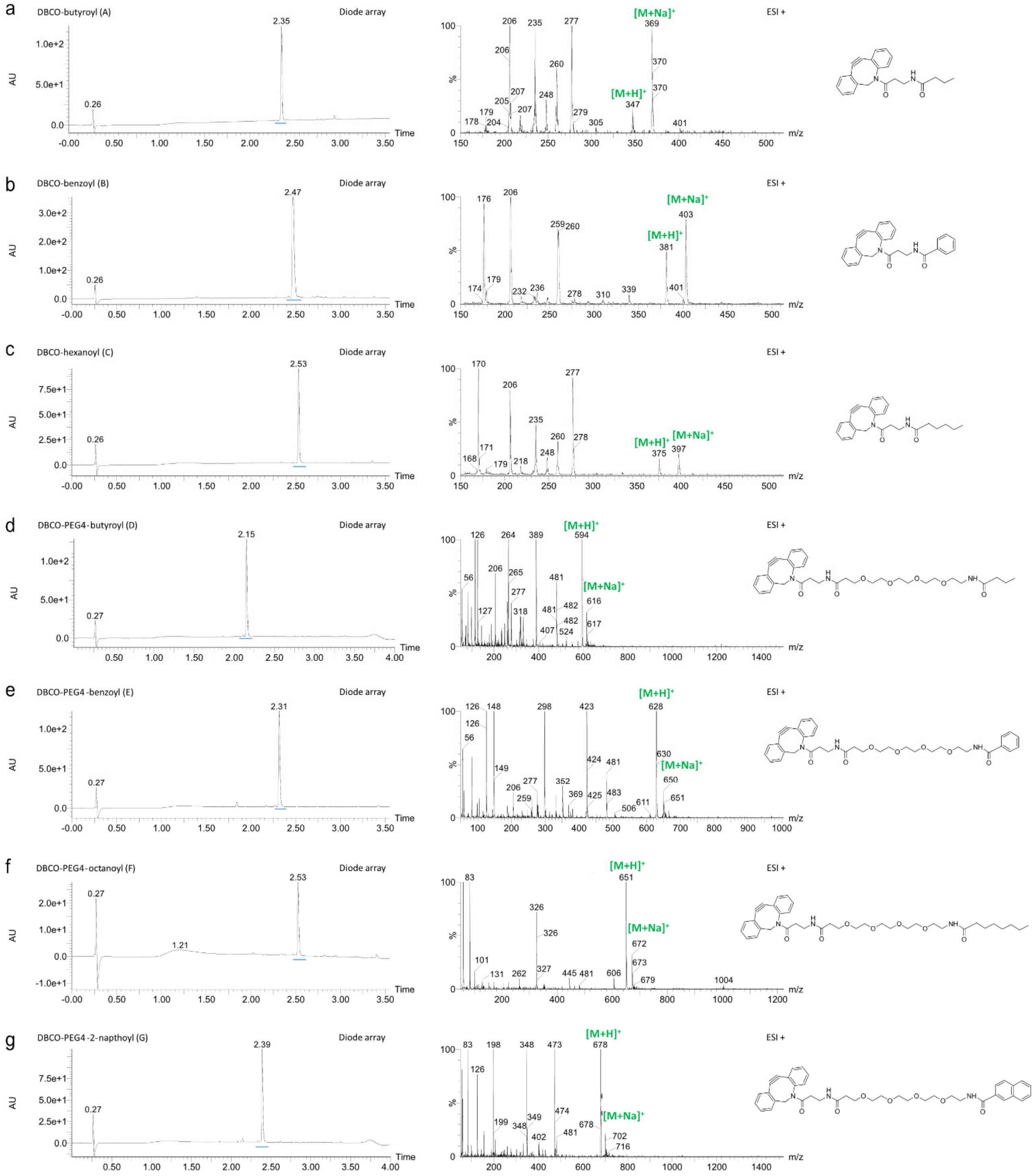

Figure S2. UPLC-MS analyses of hydrophobic moieties.

Corresponding peaks are annotated in green. Diode array: 190-800 nm.

a) MS m/z $C_{22}H_{22}N_2O_2$ $[M+H^+]^+$ calculated: 347.2, found: 347.2; $[M+Na^+]^+$ calculated: 369.2, found: 369.2.

b) MS m/z $C_{25}H_{20}N_2O_2$ $[M+H^+]^+$ calculated: 381.15, found: 381.4; $[M+Na^+]^+$ calculated: 403.15, found: 403.1.

c) MS m/z $C_{24}H_{26}N_2O_2$ $[M+H^+]^+$ calculated: 375.2, found: 375.4; $[M+Na^+]^+$ calculated: 397.2, found: 397.6.

d) MS m/z $C_{33}H_{43}N_3O_7$ $[M+H^+]^+$ calculated: 594.3, found: 594.4; $[M+Na^+]^+$ calculated: 616.3, found: 616.4.

e) MS m/z $C_{36}H_{41}N_3O_7$ $[M+H^+]^+$ calculated: 628.3, found: 628.4; $[M+Na^+]^+$ calculated: 650.3, found: 650.3.

f) MS m/z $C_{37}H_{51}N_3O_7$ $[M+H^+]^+$ calculated: 650.4, found: 650.4; $[M+Na^+]^+$ calculated: 672.4, found: 672.4; $[M+2H^+]^{2+}$ calculated: 325.7, found: 325.7.

g) MS m/z $C_{40}H_{43}N_3O_7$ $[M+H+]^+$ calculated: 678.3, found: 678.5; $[M+Na^+]^+$ calculated: 700.3, found: 700.4.

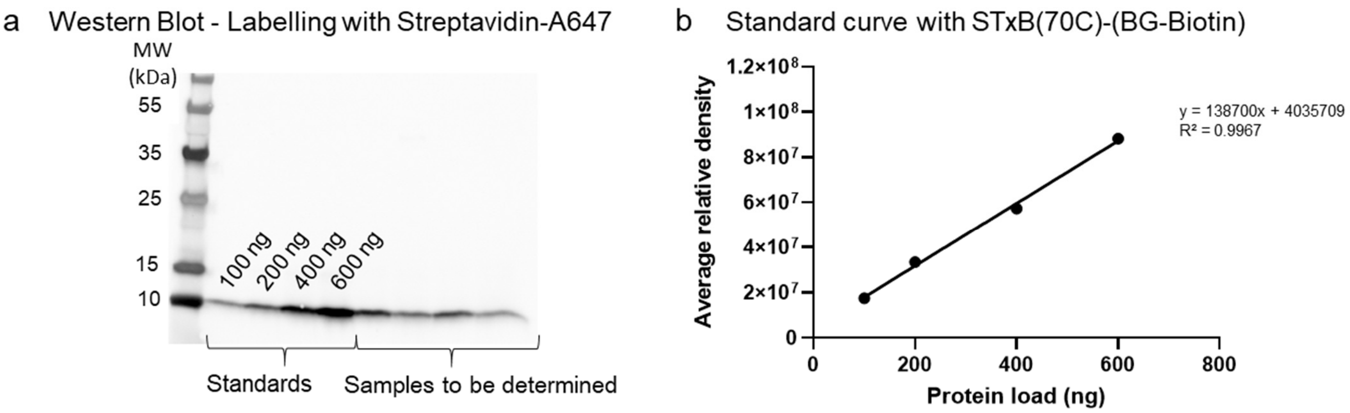

Figure S3. Quantification of the concentrations of STxB double conjugates by western blotting.
STxB double conjugate concentrations were determined using the biotin from the translocation reporter to react with streptavidin. a) Membrane for the concentration assessment of the conjugates by Streptavidin-Alexa647 western blot. b) Standard curve with STxB(70C)-(BG-biotin) from 100 ng to 600 ng.

Table S1. Double conjugation yields for coupling of hydrophobic moieties and BG-Biotin to STxB double variants. Capital letters correspond to the hydrophobic moieties presented Figure 2. The yield represents the amount of conjugated protein recovered after purification over the amount of protein used for the reaction.

	A	B	C	D	E	F	G	DBCO-NH_2	DBCO-PEG4-NH_2
STxB(D3KN_3)	10.86	0,90	-	33.59	10.20	-	-	-	-
STxB(E10KN_3)	13.43	1.07	-	77.63	16.36	-	-	-	-
STxB(H58KN_3)	15.40	4.28	-	-	-	-	-	-	-
STxB(N59KN_3)	9.51	2.83	1.63	62.42	9.22	8.06	1.05	12.44	42.78
STxB(N59K-PEG4-N_3)	15.62	4,17	6.89	-	-	-	-	-	-

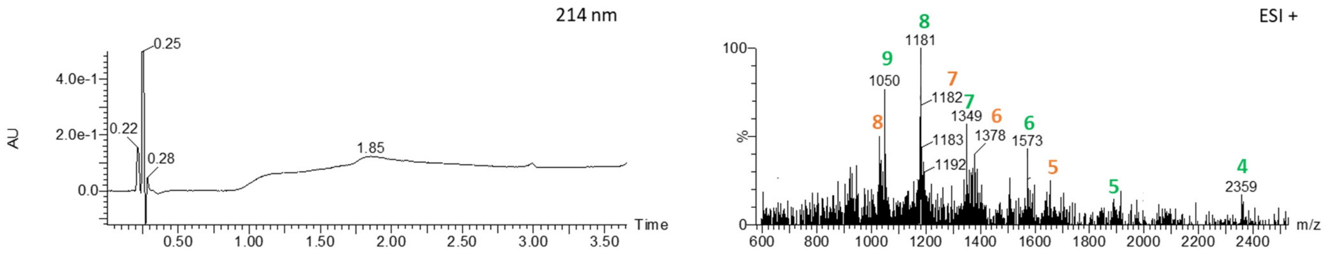

Figure S4. UPLC-MS analysis of STxB(N59KN_3)(70C) conjugated to DBCO-benzoyl and BG-Biotin.
Peaks corresponding to the expected product are annotated with their corresponding positive charge in green. Peaks annotated in orange correspond to monomers with a *t*-butyl adduct on the additional cysteine. MS m/z $C_{424}H_{635}N_{111}O_{124}S_5$ $[M+4H^+]^{4+}$ calculated: 2358.9 $[M+5H^+]^{5+}$ calculated: 1887.3; $[M+6H^+]^{6+}$ calculated: 1572.9; $[M+7H^+]^{7+}$ calculated: 1348.4; $[M+8H^+]^{8+}$ calculated: 1180.0; $[M+9H^+]^{9+}$ calculated: 1049.0.

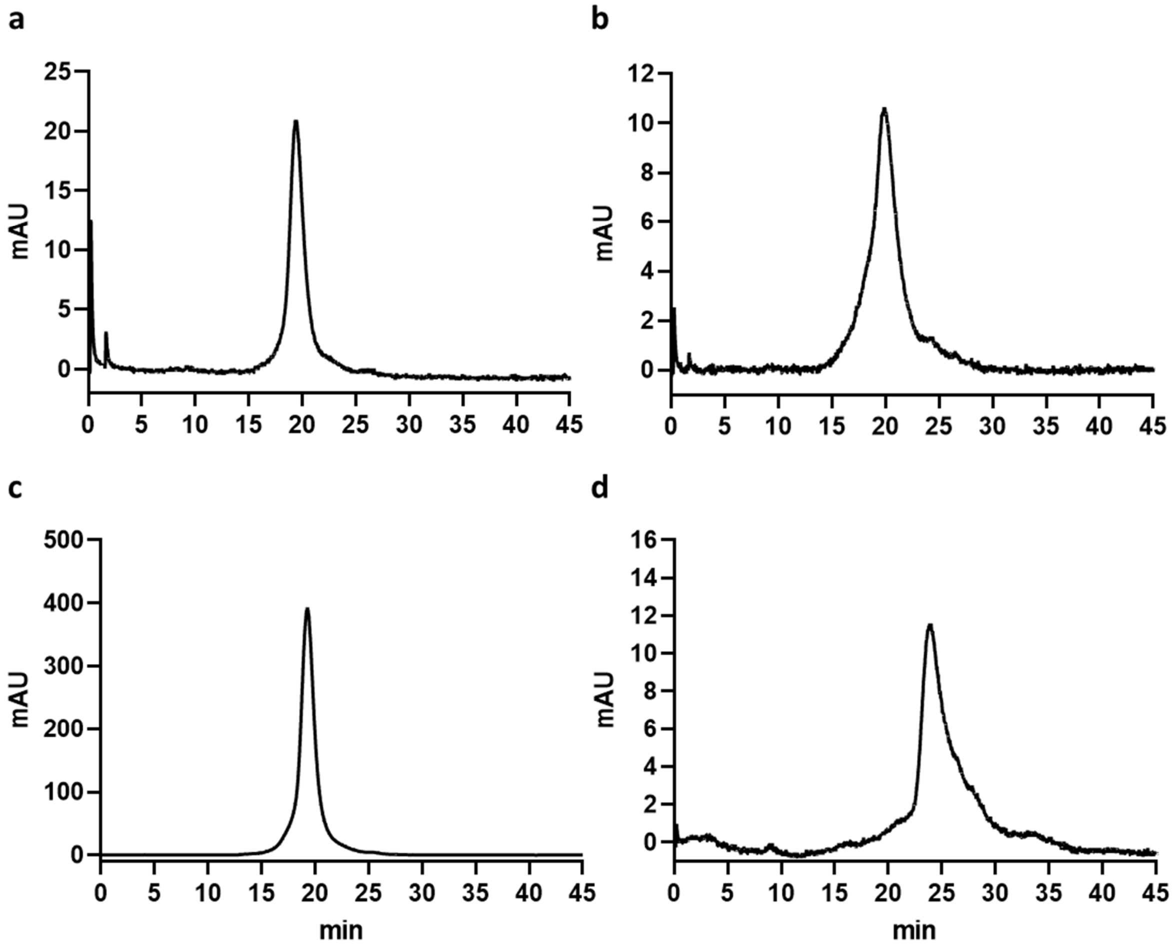

Figure S5. Size exclusion chromatography with STxB variants.

mAU is measured at 280 nm. Chromatogam of: a) Recombinant wildtype STxB, b) synthetic wildtype STxB, c) STxB(N59KN$_3$)(70C), d) STxB(N59KN$_3$)(70C) conjugated to DBCO-benzoyl.

Table S2. Size exclusion chromatography retention times for STxB variants.

Retention times for synthetic wildtype STxB and for the double variant STxB(N59KN$_3$)(70C) are similars to that of recombinant wildtype STxB. The delay for the retention time of the STxB(N59KN$_3$-DBCO-benzoyl)(70C) conjugate might be due to hydrophobic interaction with the column.

	Peak (min)
Recombinant STxB-WT	19.44
Synthetic STxB-WT	19.89
STxB(N59KN$_3$)(70C)	19.26
STxB(N59KN$_3$-DBCO-benzoyl)(70C)	23.97
